# A mixed-methods evaluation of the uptake of novel differentiated ART delivery models in a national sample of health facilities in Uganda

**DOI:** 10.1371/journal.pone.0254214

**Published:** 2021-07-22

**Authors:** Henry Zakumumpa, Kimani Makobu, Wilbrod Ntawiha, Everd Maniple

**Affiliations:** 1 Makerere University, School of Public Health, Kampala, Uganda; 2 Kenya Medical Research Institute-Wellcome Trust Research Programme, Kilifi, Kenya; 3 National Social Security Fund, Kampala, Uganda; 4 Kabale University, School of Medicine, Kabale, Uganda; University of Ghana College of Health Sciences, GHANA

## Abstract

**Introduction:**

Since 2017, Uganda has been implementing five differentiated antiretroviral therapy (ART) delivery models to improve the quality of HIV care and to achieve health-system efficiencies. Community-based models include Community Client-Led ART Delivery and Community Drug Distribution Points. Facility-based models include Fast Track Drug Refill, Facility Based Group and Facility Based Individual Management. We set out to assess the extent of uptake of these ART delivery models and to describe barriers to uptake of either facility-based or community-based models.

**Methods:**

Between December 2019 and February 2020, we conducted a mixed-methods study entailing a cross-sectional health facility survey (n = 116) and in-depth interviews (n = 16) with ART clinic managers in ten case-study facilities as well as six focus group discussions (56 participants) with patients enrolled in differentiated ART models. Facilities were selected based on the 10 geographic sub-regions of Uganda. Statistical analyses were performed in STATA (v13) while qualitative data were analysed by thematic approach.

**Results:**

Most facilities 63 (57%) commenced implementation of differentiated ART delivery in 2018. Fast Track Drug Delivery was the most common facility-based model (implemented in 100 or 86% of health facilities). Community Client-Led ART Delivery was the most popular community model (63/116 or 54%). Community Drug Distribution Points had the lowest uptake with only 33 (24.88%) facilities implementing them. By ownership-type, for-profit facilities reported the lowest uptake of differentiated ART models. Barriers to enrolment in community-based models include HIV-related stigma and low enrolment of adult males in community models.

**Conclusion:**

To the best of our knowledge this is the first study reporting national coverage of differentiated ART delivery models in Uganda. Overall, there has been a higher uptake of facility-based models. Interventions for enhancing the uptake of differentiated ART models in for-profit facilities are recommended.

## Introduction

Globally, the implementation of the universal ‘test and treat’ policy has dramatically increased the population in need of anti-retroviral therapy (ART) [1]. In order to meet the escalating demand for ART in resource-limited settings, adaptations in traditional HIV service delivery models have become a global health imperative [2–4]. Differentiated service delivery (DSD) is a novel adaptation to traditional HIV service delivery models. DSD was endorsed by the World Health Organization (WHO) in 2016 and major global HIV donors such as the President’s Emergency Plan for AIDS Relief (PEPFAR) and The Global Fund with the aim of improving the quality of HIV care and patient outcomes [5]. Additionally, DSD aims at improving health-system and programme efficiencies thereby allowing for an expansion in ART coverage. DSD has been defined as *‘a client-cantered approach that simplifies and adapts HIV services across the cascade*, *in ways that both serve the needs of people living with HIV better and reduce unnecessary burdens on the health system’* [2] DSD is said to comprise some basic elements or ‘building blocks’ [3]. These include appointment spacing of between 3 to 6 months for stable patients, out-of-facility care, and task shifting to non-clinician cadres [3]. DSD holds the immense promise of reducing overcrowding and the heavy workloads common at points of care in countries with a high HIV burden [1–3].

Since 2016, several countries with a high HIV burden in sub Saharan Africa (SSA) have been implementing national DSD scale-up programmes. These countries include Malawi, South Africa, Zimbabwe and Zambia [6–10].

**Fig 1:**
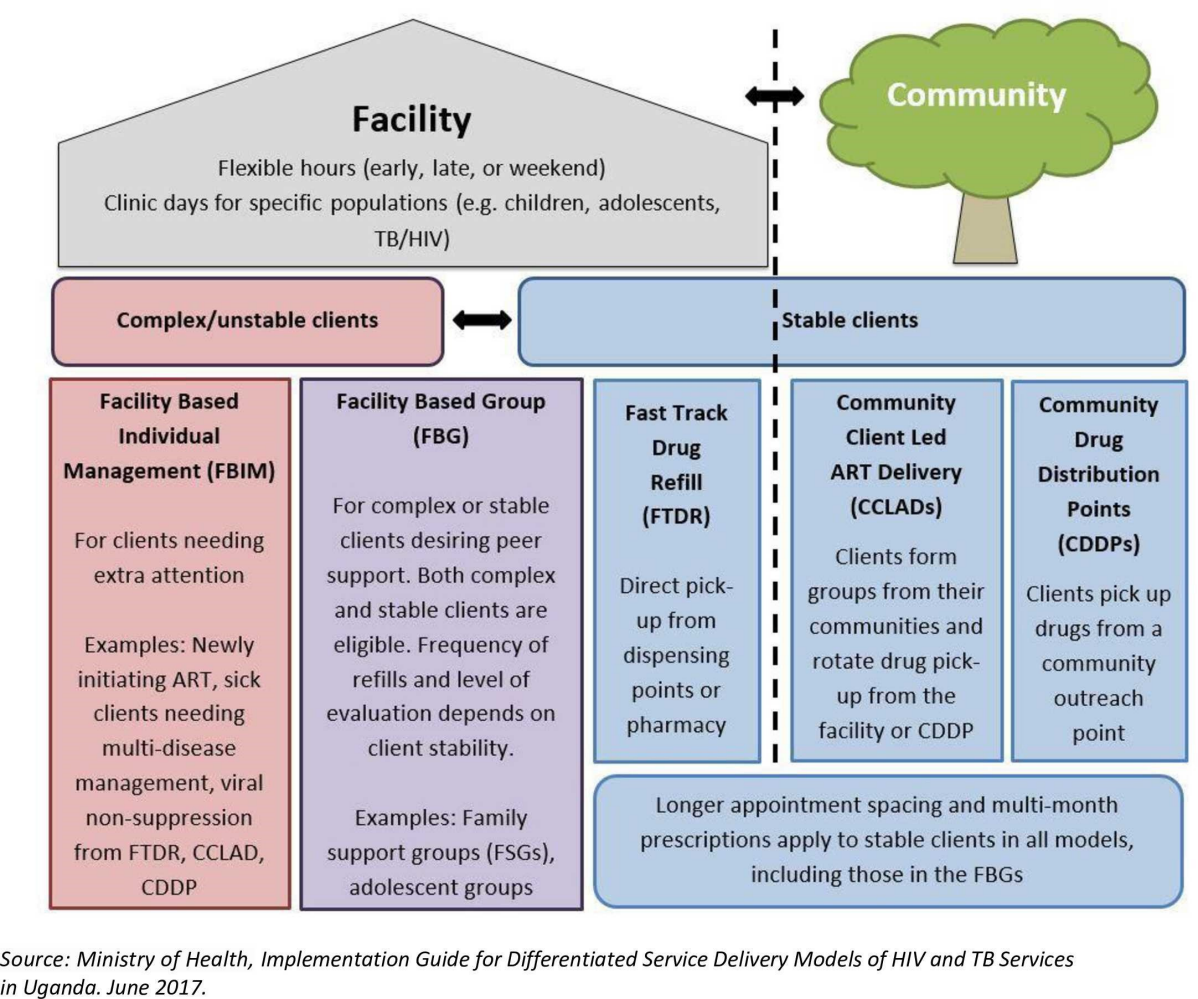
The five differentiated ART models in Uganda.

In Uganda, the national ART treatment guidelines of 2016 were updated to provide for differentiated HIV services [4]. Since 2017, the Uganda Health ministry has been implementing differentiated ART delivery models. Uganda has been implementing five models for the over 1.2 million Ugandans accessing ART [4]. **Fig 1** shows the five models currently in implementation in Uganda. Of these, four are less-intensive models catering for stable patients. These include; i) Fast Track Drug Refill (FTDR) that entails receiving 3–6 monthly ART refills-only, ii) Community Client-Led ART Delivery (CCLAD) where voluntary groups of six patients rotate in picking up ART medicines refills for each other from facilities iii) Community Drug Distribution Points (CDDPs) where outreach sites within communities are designated for ART refill pick-ups and iv) Facility Based Group (FBG) where patients form adherence support clubs [4]. Facility-Based Individual Management (FBIM) is synonymous with the default traditional facility-based model of HIV care that was undifferentiated to the needs of individual patients. Enrolling patients in less-intensive treatment models is a top priority in over-burdened health systems in SSA including Uganda [8].

It is unclear what the uptake of the various differentiated ART delivery models has been at health facilities across Uganda [11]. Although there is a steadily emerging evidence base on early implementation experiences of the roll-out of DSD for ART in countries with a high HIV burden, most of these have been experimental studies utilizing a small sample of facilities [6,7,10]. However, data on the national coverage of these novel service delivery models such as the proportion of health facilities implementing differentiated ART models are sparse [11–13]. Understanding the service coverage of differentiated ART delivery models is vital in HIV programming and planning in terms of identifying gaps in service coverage, assessing demand for particular differentiated ART delivery models and understanding patient preferences as well as addressing implementation barriers to enable further DSD scale-up [1,11]. These data are essential to inform decision making by national-level HIV program managers, funders, providers and frontline health workers in countries rolling out DSD [13]. Additionally, DSD has gained an increased importance in the context of the Covid-19 pandemic with a number of countries leveraging these models to deliver six-month ART refills to patients held up by ‘lockdown’ measures for averting spread of Covid-19. Several studies have called for assessments of the uptake of DSD in countries implementing national scale-up programs [1,5,11,12]. We set out to assess the extent of uptake of novel differentiated ART delivery models at health facilities in Uganda and to qualitatively explore reasons for variations in uptake of facility-based versus community-based models.

## Materials & methods

### Research design

We utilized a mixed-methods convergent parallel design [14,15]. We conducted a cross-sectional health facility survey to assess the extent of uptake of differentiated ART delivery models in a national sample of health facilities across Uganda. In addition, we conducted a qualitative multiple case-study of ten health facilities to explore reasons for variations in uptake of facility-based versus community-based models. This is because we aimed to understand the uptake of the DSD models from the perspectives of patients and providers given their contexts and health-system setting(s) [16]. In alignment with the convergent parallel design, the quantitative and qualitative data are presented alongside each other rather than as separate components [14,15].

### Analytical framework

In this study, we adopted a guiding analytical framework developed by Levesque and colleagues [16] which proposes a multi-level lens within which to understand factors influencing patient access to health care. This framework proposes that factors influencing patient access to health care are nested at the individual-level (e.g. ability to pay, social status), at the meso-level (location of facilities, costs) at the macro-level (health system responsiveness e.g. enabling policies and financing mechanisms) and the broader environment (such as the community setting) and that these levels are intersecting. Hence, patient access to health care is embedded within these underpinning contexts that interact in dynamic ways.

### Study sites and sample selection

#### Phase I

The units of study were Ministry of Health-accredited ART-providing facilities in Uganda [17]. We applied a multi-stage stratified cluster sampling design procedure as we sought to construct a nationally representative sample of health facilities in Uganda. Districts, from which facilities were selected, were grouped by the ten geographic sub-regions of Uganda as defined by the Uganda Bureau of Statistics [18]. We randomly sampled one district per region by urban and rural strata. The second step entailed sampling health facilities from each of the selected districts. Sampled health facilities included regional referral hospitals, district hospitals and health centres (IV and III). Ten districts were selected through the district sampling frame (five rural and five urban), Kish’s formula [15] was applied to generate a sample of 195 health facilities (from a universe of N = 394) through a facility sampling frame: a) Inclusion of all regional referral and general hospitals who would offer consent b) **Table 1** shows that we sought a sample of Health Centres IVs and IIIs based on Probability Proportional to Size (PPS) sampling based on a published Ministry of Health report [17,19]. Overall, 116 health facilities agreed to participate in the study giving us a response rate of 59.48%.

**Table 1:** Sampling proportional to size by ART service delivery characteristics.

Level	%
Regional Referral Hospital	4
General Hospital	24
Health Centre IV	33
Health Centre III	13
Health Centre II	1
Research and specialized clinics	9
Private, for-profit	17
	100

Source: Ministry of Health: The status of Antiretroviral Therapy service Delivery in Uganda [17].

#### Phase II

We adopted a qualitative multiple case-study design [20]. We purposively selected one health facility in each of the 10 sub-regions of Uganda [18]. Table 2 shows the demographic characteristics of participants at case-study facilities. The qualitative phase of the study aimed at gaining an in-depth understanding of the reasons for variations in uptake between facility-based and community-based differentiated ART delivery models [20]. Furthermore, we purposively selected health facilities which had at least a one-year experience of implementing community-based models. For instance, due to the dearth of facilities implementing the Community Drug Distribution Points model, we purposively included PNFP-007, as a case-study facility.

**Table 2:** Category of participants in qualitative arm of the study.

Category	Number of participants
1. ART clinic managers	n = 16
2. National-level HIV program managers	n = 8
3. District Health Teams	n = 10
3. Recipients of HIV care (six FGDs)	n = 56

### Data collection

#### Phase I: Quantitative data collection

A standardized, structured questionnaire was fielded to the ART clinic manager in each of the 116 health facilities. Our paper-based, 35-item questionnaire was researcher-administered, on-site, at each of the participating health facilities. Our questionnaire was pilot-tested among 12 ART clinic managers of health facilities in Mityana District in central Uganda. The 12 facilities were outside the study sample. Data were collected between September and December 2019.

#### Phase II: Qualitative data collection

We conducted sixteen in-depth face-to-face interviews (IDIs) with ART clinic managers and their staff on-site at the ten case-study facilities to understand facility-level operational contexts [21] that potentially influenced uptake or demand for either facility-based or community-based models. We selected at least one ART clinic manager from each of the case-study facilities.

#### Focus group discussions

We aimed to understand multiple patients’ experiences of differentiated ART models as a group rather than as individuals and as such, focus groups were deemed appropriate for the purpose [22,23]. To this end, we conducted six focus group discussions (FGDs) with fifty-six patients enrolled in differentiated ART models. Examples of questions posed to patients in the focus groups include; *1*. *What do you like about being in the differentiated ART model in which you are*? *2*. *What could be changed to make the CCLAD model better*? *3*. *Ideally*, *how would you like to get your ART medication*?. We conducted at least one FGD with patients enrolled in each of the five differentiated ART delivery models while aiming for a balance between community-based (three FGDs) and facility-based models (three FGDs) currently in implementation in Uganda. Three FGDs were conducted with patients enrolled in community-based models (Fig 1) for at least a year. We conducted three FGDs with patients enrolled in each of the three facility-based models for at least one year (Fig 1). Each of the six FGDs comprised at least eight participants of mixed-gender groups.

ART clinic managers at case-study sites were asked to support recruitment of patients enrolled in differentiated ART models based on the inclusion criteria: Recipients of HIV care who were 18 years or older who had been enrolled in a differentiated ART delivery model for at least twelve months. We excluded receipts of HIV care who were below 18 years of age and those who had been enrolled in a differentiated ART model for less than twelve months.

The focus groups were audio-recorded with the permission of the FGD participants. The first author was assisted by four research assistants. The research assistants observed the participants, took notes and operated the recorder. Data were collected between December 2019 and February 2020.

Furthermore, to gain a national-level perspective on the roll-out of differentiated ART models across Uganda, we conducted eight Key Informant Interviews (KIIs) with national-level HIV program managers. To gain a sub-national level lens on DSD implementation, we interviewed ten district health team leaders overseeing case-study facilities. **Table 2** shows the category of participants in the qualitative arm of the study.

The qualitative interviews were conducted by the first author who holds a PhD in health policy and systems research with an academic background in the social sciences and qualitative research [29,37].

### Ethical clearance

This research received ethical approval from Mildmay Uganda Research Ethics Committee (MUREC) under instrument: REC REF 0408–2019. MUREC is accredited by the Uganda National Council of Science and Technology (UNCST). All participants signed a written consent form before participating in the study.

### Data analysis

#### Phase I: Quantitative data

Data generated from the health facility survey instrument were cleaned, edited and initially entered into EpiData software (version 3.1) (EpiData Association, http://www.epidata.dk/) and later exported into STATA (version 13) (College Station, TX, USA) for descriptive analyses that included frequency counts and percent distributions. Cross tabulations were used to investigate associations between uptake of differentiated ART models (outcome measure) and selected explanatory variables (such as ownership-type of facility (public/private), setting (rural/urban), HIV client load (high volume/low volume). The level of statistical significance using *p*-values was set at *p* < 0.05.

#### Phase II: Qualitative data

Qualitative data were analysed by thematic approach following the procedures recommended by Miles & Huberman [22]. The audio-recorded interviews and focus group discussions were transcribed verbatim. Our qualitative data analysis approach followed an iterative process [22]. We followed four major steps in data analysis: a) *Data familiarization*: As a first step, the transcripts were read multiple times (by HZ, WN & KM) b) *Development of a coding framework*: In the second stage, a descriptive coding scheme was inductively generated by HZ, WB & EM from multiple readings of the interview transcripts. Additional codes and sub-codes were developed based on themes explored in the interviews (such as the benefits of being in certain models; for instance, savings in time and transport costs for those enrolled in community-based models) and challenges (such as reduced physical interaction with health workers) as well as those proposed in our adopted analytical framework [16]. The joint codebook was validated by a 4^th^ reader. The same coding structure was applied for both interviews with health workers and focus groups with patients. c) *Abstraction of coded data into thematic categories*: In the third stage, the emergent codes were then abstracted into thematic matrices [23] with the aid of Atlas.ti software. The team of investigators conducted weekly meetings during the period of data analysis, whereby themes were developed, revised, and refined. For instance, themes around preference for facility-based models included the psycho-social satisfaction derived from being seen regularly by a health worker and the opportunity for comprehensive health care (such as the management of opportunistic infections). d) *Interpretation and overall synthesis*: The fourth stage involved overall interpretation and synthesis involving all the authors [23].

### Mixed-methods integration

Our quantitative and qualitative data were merged during the phase of overall interpretation and synthesis of study findings [14,24,25]. To this end, findings from each of the two data sets were placed side by side as we sought convergence in answering the study objective [15]. The quantitative and qualitative data are presented alongside each other under the emergent sub-themes described in the “Results” section.

## Results

### Characteristics of participating health facilities

Overall, 116 health facilities across Uganda were included in the study. In terms of ownership-type, the majority 68 (58.62%) were public facilities while 27 (23.27%) were private not-for-profit (PNFP) and 21 (18.10%) were private for-profit (PFP).

With regard to level of care in the Ugandan health system [17], most participating facilities (34/116 or 34.46%) were Health centre IVs (sub-district facilities) followed by Health centre IIIs (sub-county) (31/116 or 31.36%) and general hospitals were 24 (24.55%).

By setting, the majority of health facilities (69 or 61.69%) were based in urban settings compared to 43 (38.39%) health facilities located in a rural setting.

In terms of HIV client loads, the majority of health facilities (75/116 or 68.81%) had at least 500 or more active ART clients compared to 34 (31.19%) health facilities which reported having less than 500 active ART patients.

### Characteristics of respondents

A total of 116 ART clinic managers participated in this study. In terms of gender, 51% (59/116) were males while 49% (57/116) were females. More than a half of all respondents (54%) were in the 30–35 age range.

The overall mean work experience of respondents was 8 years (1–20) with a (4.1) standard deviation

In terms of health worker cadres, Clinical Officers (n = 40, 34.1%) were the most represented, followed by nurses (n = 37, 31.8%) and physicians (n = 24, 21.1%). Clinical Officers are a category of health workers in Uganda below the rank of physicians. Clinical Officers undertake three years of post-secondary school training in a tertiary, non-university, training institution.

### Year when differentiated ART models were first implemented

**Fig 2:**
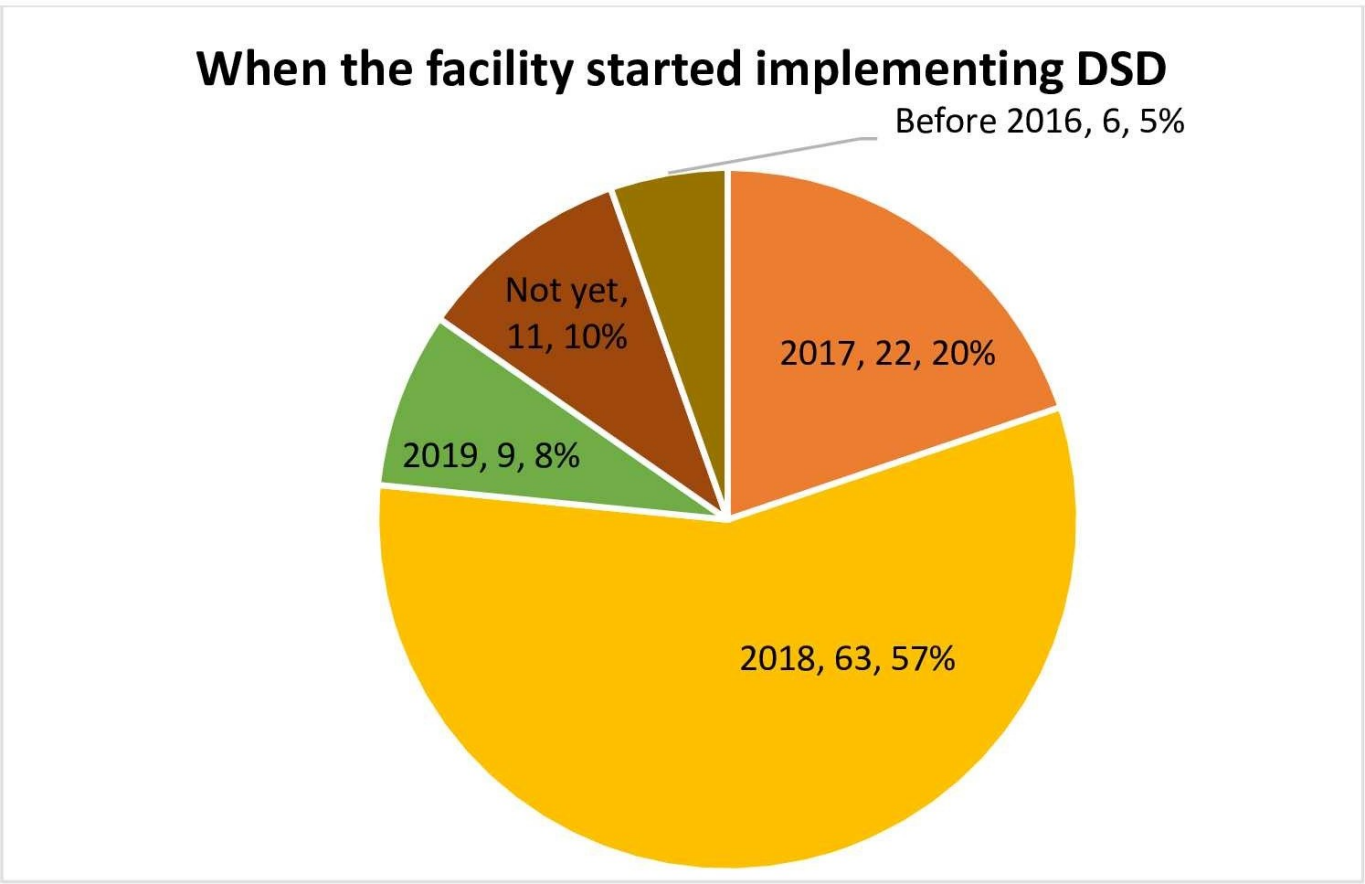
Year when differentiated ART delivery was first implemented.

**Fig 2** shows that most of the health facilities (63/116 or 57%) commenced implementation of differentiated ART models in 2018. However, our qualitative data from facilities implementing these models revealed that ART clinic managers perceived differentiated ART delivery as not an entirely new service delivery innovation and that they had been informally implementing some models (such as 3-monthly appointment spacing) prior to the release of formal national ART guidelines designating them as such in 2016.

*‘We were already doing differentiated service delivery even before the term was coined as such. That term (DSD) came in around 2014 but DSD has been with us for such a long time. We were already doing three- month refills long before this became part of formal guidelines or a systematic way of implementing it was developed. DSD is not new. What is new is the terminology used.’*

*[ART clinic in-charge, PNFP-001]*

### Proportion of facilities implementing differentiated ART delivery models

**Fig 3:**
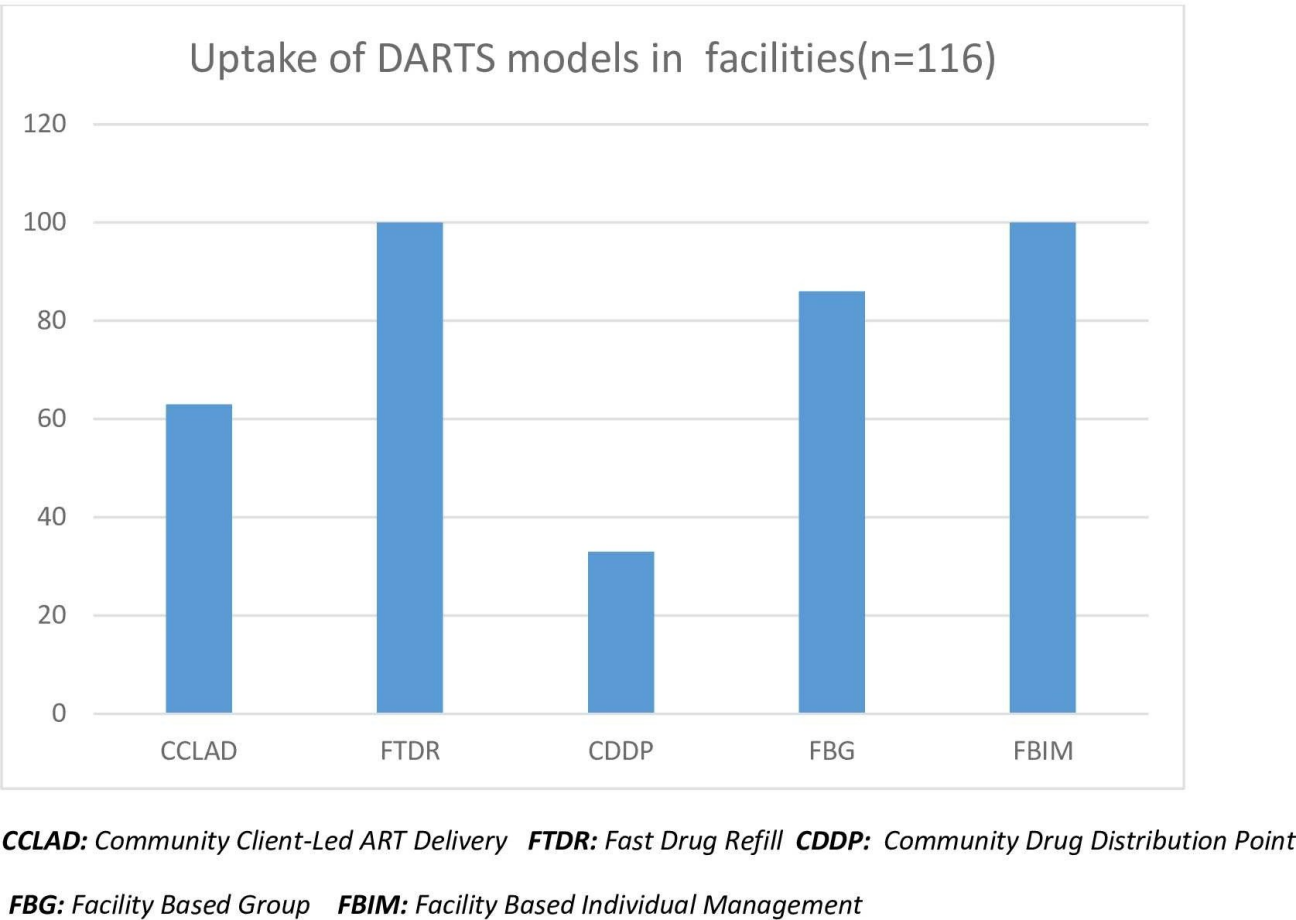
Uptake of differentiated ART delivery models.

The majority of health facilities 100 (86%) were implementing at least one differentiated ART delivery model. As **Fig 3** shows, there has been a higher uptake of facility-based models when compared to community-based ones. The most implemented facility-based differentiated ART model was the Fast Track Drug Refill (FTDR) model. FTDR was reported to be in implementation in 100 (86%) of health facilities in our sample.

Community Client-Led ART Delivery (CCLAD) was the most popular community-based model implemented in more than a half of all health facilities 63 (54%). Overall, Community Drug Distribution Points (CDDP) had the lowest uptake at (n = 33, 24.88%) of all differentiated ART delivery models.

Across our in-depth interviews with ART clinic managers it was revealed that the CDDP model was widely perceived as costly to implement. While the CCLAD model entailed self-forming patient groups primarily financed by contributions from individual members such as sharing transport costs for picking ART refills, the Community Drug Distribution Points (CDDPs) model depended substantially on the resource envelop of the hosting health facility. Donors supporting the CDDP model in some participating facilities were reported to have discontinued funding due to escalating operational expenses. The costs required for running CDDPs were identified. These include fuel for transporting ART refills into communities, monetary field allowances for facility-personnel during visit to communities and the costs of maintaining the physical infrastructure in the community hosting the drug pick-up points.

*‘‘ We have some community approaches that are expensive such as CDDPs because the hospital has to foot the transport costs of ferrying ART refills into rural, remote outreach posts. With the CCLAD model there is no added expense on the health system. It is the patients meeting the costs of transport for picking ART refills from hospitals” [National-level HIV program manager, Ministry of Health]*.

High-volume sites or those with 500 active ART clients or more, were implementing the CCLAD model more than low-volume HIV clinics or sites which had less than 500 active clients (p < 0.001).

### Characteristics of facilities implementing all five differentiated ART models

Overall, only 25 facilities (21.55%) were implementing all five differentiated ART models recommended by the Ministry of Health of Uganda. The characteristics of the 25 health facilities are represented in **Table 3**. This table shows that almost half (48%) of the facilities reporting implementation of all five differentiated ART models were private not-for-profits (PNFPs) the majority of which had a faith-based foundation. More than half of the 25 health facilities were general hospitals while a quarter of them were Regional Referral Hospitals (RRHs). This may suggest that the uptake of differentiated ART delivery models is highest at the most advanced level of care in the Ugandan health system (especially at the tertiary level).

**Table 3:** Characteristics of health facilities implementing all five DARTS models.

	ACRONYM	OWNERSHIP-TYPE	LEVEL OF CARE IN UGANDAN HEALTH SYSTEM	SETTING	GEOGRAPHIC SUB-REGION [11]	CUMMULATIVE ART PATIENT LOAD (As of September 2019)
1	PUB-001	PUBLIC	Referral Hospital	Urban	Southwest	11,026
2	PUB-002	PUBLIC	Referral Hospital	Urban	Western	4,542
3	PUB-003	PUBLIC	Referral Hospital	Urban	West Nile	6,700
4	PUB-004	PUBLIC	Referral Hospital	Urban	North	12,495
5	PNFP-001	NOT FOR PROFIT	Referral Hospital	Urban	North	5,447
6	PUB-005	PUBLIC	General Hospital	Urban	Western	2,610
7	PUB-006	PUBLIC	General Hospital	Rural	Central 2	1,549
8	PUB-007	PUBLIC	General Hospital	Rural	West Nile	2,402
9	PUB-008	PUBLIC	General Hospital	Rural	Central 2	5,349
10	PUB-009	PUBLIC	General Hospital	Rural	Eastern	769
11	PNFP-002	NOT FOR PROFIT	General Hospital	Urban	East Central	7,452
12	PNFP-003	NOT FOR PROFIT	General Hospital	Urban	Kampala	1084
13	PNFP-004	NOT FOR PROFIT	General Hospital	Rural	Western	3,518
14	PNFP-005	NOT FOR PROFIT	General Hospital	Peri-urban	West Nile	1,035
15	PNFP-006	NOT FOR PROFIT	General Hospital	Peri-urban	Southwest	2,603
16	PNFP-007	NOT FOR PROFIT	General Hospital	Peri-urban	North	8,003
17	PNFP-008	NOT FOR PROFIT	General Hospital	Peri-urban	Central 1	1,070
18	PNFP-009	NOT FOR PROFIT	General Hospital	Urban	Central 1	3,542
19	PUB-010	PUBLIC	Health centre IV	Peri-urban	Central 1	1,078
20	PUB-011	PUBLIC	Health centre IV	Rural	Central 1	1,243
21	PUB-012	PUBLIC	Health centre IV	Rural	Southwest	1,780
22	PUB-013	PUBLIC	Health centre IV	Peri-urban	Central 1	1,586
23	PUB-010	NOT FOR PROFIT	Health centre IV	Rural	Central 2	2,006
24	PNFP-011	NOT FOR PROFIT	Health Centre IV	Rural	Central 1	2,578
25	PNFP-012	NOT FOR PROFIT	Health Centre III	Urban	Central 1	651

### Uptake of models by ownership-type of health facility

We found variations in uptake of differentiated ART delivery models by ownership-type of a health facility. Overall, Private for-profit (PFP) health facilities reported the lowest uptake of differentiated ART models across ownership-type of facility. Out of the 21 private for-profit facilities in our sample, only a third had implemented differentiated ART models at all. In contrast, 67% of all public and 52% of all Private not-for-profit (PNFPs) commenced implementation of differentiated ART delivery models in the year 2018 alone. The year a health facility started implementing differentiated ART models and ownership-type of health facility are significantly related (p = 0.001).

Our qualitative data provided insights into why there was relatively low uptake of differentiated ART models in private for-profit facilities. Participants from for-profit facilities reported that their health workers had not been trained in differentiated ART delivery even when their counterparts in public and not-for-profit facilities were being trained by PEPFAR implementing organizations and the Uganda government at no charge as highlighted in the quote below:

*‘No training in differentiated HIV services has been done for our staff in private hospitals. They always tell our staff to pay when attending training by the Ministry of Health. Private hospitals have to dig into their pockets to pay for training of health workers yet this is free for public and not-for-profits. But we don’t have a budget for that’ [ART clinic in-charge, PFP-06]*.

Interviews with national-level HIV program managers at the Uganda Ministry of Health and district health team leaders revealed that donors in Uganda prioritized public and private-not-for-profit (PNFP) facilities because these had the highest HIV client loads and hence donors perceived their investments to have ‘a higher yield’ there. As such, it emerged from this study that for-profit (PFP) facilities were not being prioritized in the national scale-up of differentiated ART delivery across Uganda and that health workers in this category of health facilities had not been trained on how to offer DSD models.

*‘Yes, it is true that our regional PEPFAR implementing organization has not included for-profit facilities in their DSD scale-up drive. Even their health workers have not been invited in the on-going trainings to orient them in differentiated HIV services’ [District Health Officer, Eastern Uganda]*.

High-volume facilities (those with 500 or more active ART clients) implemented a more diverse mix of models across both community (mainly CCLAD) and facility-based (principally FTDR) models compared to low-volume facilities (those with less than 500 active ART clients) which exclusively implemented facility-based (principally FTDR) models (p = 0.001).

### Understanding the relatively low uptake of community-based models

**Fig 4:**
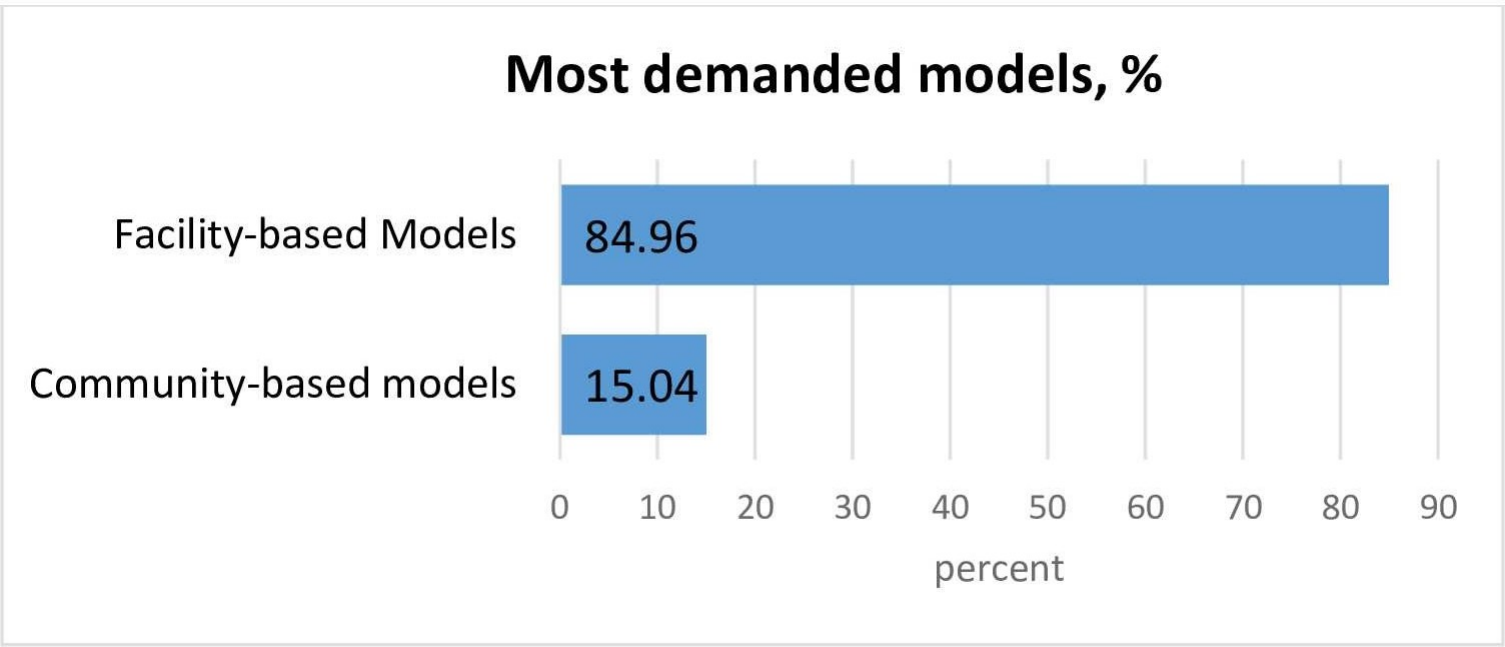
Demand for differentiated ART models.

Overall, the uptake of community-based models was less than that of facility-based models. **Fig 4** shows that facility-based models have received more demand from patients when compared to community-based models according to the majority of respondents (85.0%).

Our qualitative findings shed light on why this was the case. Participants attributed the relatively low demand for community-based models to HIV-related stigma. Patients were said to be filled with fear of unintentional disclosure of HIV status or plainly, being known to live with HIV, by peers living in the same neighbourhoods if they joined patient groups such as CCLAD.

*‘Patients do not actually like the community models because of stigma, especially CCLAD which involves forming groups of six patients living in the same community. That means all the patients in the group know each other and reside in the same neighbourhood. But patients will tell you “I do not want the other person to know that I am sick and get (HIV) care from this facility’ [ART clinic manager, PF-03]*.

‘*Patients are aware of DSD models but the majority of them have stigma*. *They fear that their colleagues will gossip about them in the villages so some people decide not to join the groups because of that they decide to come to the facility and pick their drugs*’ *[Focus group with patients, PUB-009]*.

#### Rural-urban differences in DSD uptake

Rural-based facilities implemented the Community Client-Led ART delivery (CCLAD) model (67.4%) more than urban-based facilities (42.0%) (p < 0.001). The latter category of facilities reporting a more even uptake of both community and facility-based models (such as Fast Track Drug Refill at 44.9%).

In-depth interviews with ART clinic managers revealed that urban patients were sophisticated and that they tended to prefer individualized facility-based care due to their need for privacy and the convenience offered there.

*‘Patients in urban settings like Kampala (city) do not want to form community groups. They just want to go to the facility, get their drugs and go home, or go to a point somewhere, get their drugs and go home. Patients in towns are not interested in forming groups. They are interested in convenience and saving time. They just want to go to a nearby pharmacy, pick their drugs and in ten minutes get out’ [ART clinic manager, PUB-004]*.

Our qualitative findings also suggest that there is a section of patients who prefer regular face-to-face interactions with health workers and the psychosocial support and satisfaction derived in standard clinic-based care. There was a perception among some patients that enrolling in community-based models entails, to some degree, a dis-engagement from the formal health system which offers some insight into understanding patient preferences and the outcomes of uptake of varied differentiated ART delivery models.

*‘Community models are good because they reduce transport costs and allow us more time at work but for me, as an individual, I want more of that psychosocial service… that personal touch by a health worker. That physical interaction. Because if you are not at the facility you don’t get that (individual) attention and care and that is why I prefer to come here on a regular basis’ [Focus group with patients, PNFP,003]*.

On the other hand, several patients in rural-based facilities expressed satisfaction with community-based models owing to the savings in time in seeking facility-based care and the significant reduction in travel costs associated with being enrolled in ART refill pick- ups from outreach sites within the community (CDDPs). Patients enrolled in the CCLAD model appreciated the opportunity of sharing the burden of transport costs for ART refill pick-ups from facilities.

‘*The number one advantage I have seen with being in a (CCLAD) group is that it is cost effective in that instead of all of us coming to the hospital*, *it is only one person who comes*. *Instead of the six of us spending 60*,*000 shillings ($16*.*3) coming to the hospital*, *we contribute only 10*,*000 shillings ($ 2*.*7) from amongst ourselves to send only of us to pick our (ART) refills’ [Focus group with patients*, *PUB-003]*

#### Gender dimensions in uptake of community models

Interviews with ART clinic managers revealed gender dimensions in the uptake of community-based models. Across six case-study facilities it was consistently reported that the majority of the members of CCLAD groups were adult females and that adult males had not taken as active an interest in enrolling in CCLADS groups when compared to adult females.

*‘We are noticing some gender dynamics in uptake of community models. Most of the CCLAD groups we have here are made up of mainly women. Men have shunned these groups. The most active within these groups are actually women who are loyal to their groups and they are consistent in them’ [ART clinic in-charge, PUB-002]*.

### The most practical differentiated ART model to implement in the assessment of ART clinic managers

We asked health facility staff to mention the most practical DSD model to implement in terms of one that required the least material resources and health worker in-put at the facility-level.

**Fig 5:**
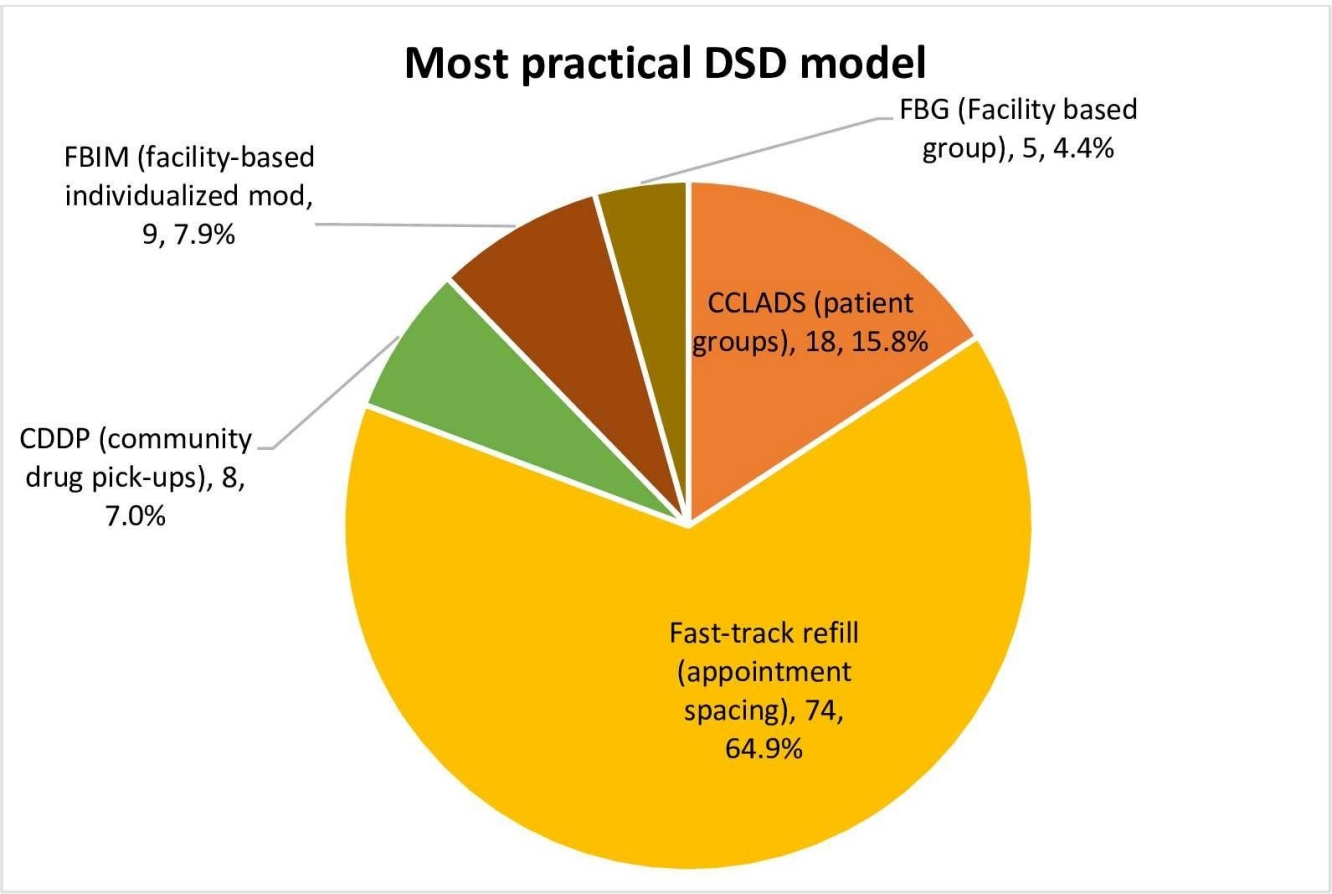
The most practical differentiated ART delivery model.

**Fig 5** shows that the majority of them (64.9%) selected Fast Track refill (FTDR) as the most practical model to implement in terms of requiring the least resource in-puts (p < 0.001).

Our qualitative data, particularly through interviews with ART clinic managers, offered keen insights into understanding why FTDR was selected as the easiest DSD model to implement.

*‘The Fast Track Drug Refill (FTDR) model has had a high success rate with us. This is good because it is the easiest to implement at the facility-level in my opinion. The guidelines are very clear, you conduct two viral load tests and if the patient is stable in the two instances, you then extend their appointments by 3–6 months. It is that easy’ [ART clinic in-charge, PNFP-002]*.

In contrast, ART clinic managers contended that community-based models (such as community drug distribution points) were relatively expensive to implement and yet facilities had not received adequate funding and resource facilitation from leading donors such as PEPFAR and the Uganda government to implement them.

## Discussion

Although differentiated ART delivery has been implemented nationally in several countries with a high HIV burden since 2016, there is a paucity of data on the extent of uptake of these novel service delivery models. We set out to assess the extent of uptake of differentiated models at health facilities in Uganda and to understand reasons for variations in uptake between facility-based and community-based models.

### Preference for facility-based models

In the present study, we found that Fast Track Drug Refill was the most implemented less-intensive DSD model in our sample of health facilities in Uganda. Additionally, the majority of ART clinic managers selected this model as the most practical to implement in terms of requiring less resource in-puts for implementation. A study in Zambia reported that visit spacing (the equivalent of FTDR in Uganda) was rated as the most important element of differentiated ART delivery by patients [9]. In our study, health facilities indicated that facility-based models had a higher demand from patients compared to community-based models. Our finding adds to the accumulating evidence base pointing to patient preferences for facility-based individualized ART services [8,10]. Another study by Rabkin and colleagues in Zimbabwe reported that patients preferred facility-based individualized care in a discreet choice experiment they conducted [8].

### Relatively low uptake of community models

Overall, our findings indicate a relatively low uptake of community-based differentiated ART models. Although previous studies point towards a trend of patient preference for facility-based HIV care [8,10,37], our qualitative findings suggest that there is an interaction between *demand-side* and *supply-side* barriers to patient enrolment in community-based models [16]. From a *demand-side* dimension, HIV-related stigma and the fear of breach of confidentiality of HIV status by patients were identified as barriers to enrolment in community models. From a *supply-side* dimension, providers indicated that PEPFAR implementing organizations in their geographic sub-regions in Uganda had not provided them with sufficient funding to enable them implement community models such as community drug distribution points (CDDPs). CDDPs may have had the least uptake due to the considerable financial and logistical investments needed to implement them and the fact that the model is intended for hard-to-reach populations such as by patients living on islands in Uganda. CDDPs were perceived to be relatively expensive to implement as they required fuel to transport health workers into the community and off-site monetary allowances. This may point to the need for further research on the relative cost-effectiveness of the varied DSD models currently in implementation in Uganda. A study by Sharer and colleagues [7] in South Africa reports that limited financial and human resources are impediments in the scale up of differentiated ART delivery models. Another study from South Africa highlights these barriers to the roll-out of community-based outreach points for ART refill pick-ups [28]. However, Community-based ART delivery mechanisms are gaining increasing importance in the context of ‘lock down’ restrictions imposed as part of Covid-19 prevention measures [26,27].

On a policy and programming note, our study reveals a need for devising interventions for stigma reduction as well community engagement with recipients of care aimed at enhancing uptake of community models through sustained sensitizations [28,29]. In addition, an interesting explanatory finding of the low uptake of community-based models is the desire by clients to see a health worker in person regularly. It suggests that HIV clients have a strong need for psychosocial support which, perhaps, was under-appreciated in the design of current differentiated ART delivery models in Uganda. There is need to enhance this aspect so that even stable patients who receive their ART refills from the community can feel confident that they are fully taken care of. Our study is one of the first to suggest a gender dimension in the uptake of differentiated ART delivery models especially with regard to the notion of adult females being more represented in voluntary patient groups of rotating ART refill pick-ups. However, gender dimensions have been previously observed, broadly, in trends in seeking HIV care and ART adherence including observations around the intersection between stigma and gender [30,31].

Our findings emphasize the need for protecting patient privacy in HIV service delivery, the need for training health workers in maintaining patient confidentiality, and the need for further community engagement drives for combating HIV-related stigma. Although there exist several health policies in Uganda relating to combating HIV-related stigma they remain largely on paper. There is an urgent need to fully operationalize these policies in order to empower people living with HIV to live positively.

### Low DSD uptake in for-profit facilities

An important finding of this study is that for-profits (PFPs) had the lowest proportion of facilities implementing differentiated ART delivery models in Uganda. Our findings revealed that leading HIV donors and the Uganda government have not prioritized for-profits in the national scale-up programs for DSD roll-out. Health workers in PFPs are not included in health worker training in differentiated ART delivery. Previous studies have highlighted the perception by leading HIV donors that public facilities constitute a better ‘investment’ option due to higher HIV client loads and therefore a better ‘yield’ in donor investments [32,33]. Our study suggests that for-profits have been left behind in the roll-out of differentiated ART delivery models across Uganda and there is a need for interventions aimed at co-opting for-profits facilities in the quest for the national scale-up of these models. Private facilities constitute more than a half of all health care providers in Uganda and they are the preferred first point of interface with the health-system for the majority of Ugandans [32,33]. Moreover, Uganda’s national policy on Public-Private Partnership for Health (PPPH) considers for-profit providers as essential partners in service delivery [34]. Therefore, there is a pragmatic need to include health workers in for-profit facilities in trainings on differentiated ART delivery, as well as on-site support supervision for successful implementation. Previous studies on differentiated ART delivery models have not included for-profit health facilities in their study populations, a gap we address in this study.

### Scope of DSD models

In this study, we found that only 25 facilities (21.55%) were implementing all the five differentiated ART delivery models recommended by the Uganda Ministry of Health. Many of the facilities with the highest uptake of these models were at the tertiary level of care in the Ugandan health system (such as Regional Referral Hospitals). This trend is suggestive of a need for remedial measures for further DSD scale-up targeting health facilities at the primary care level such as at sub-district health centres in Uganda which have high HIV client loads and where differentiated ART delivery models offer promise for decongesting points of care and reducing health worker workloads. Almost all the private not-for-profits (PNFPs) implementing community-based differentiated ART models in our sample were faith-based or mission hospitals. Our study adds to the literature recognizing the contribution of mission hospitals in strengthening community health systems in Sub-Saharan Africa [35–37].

This study had a limitation which is important to acknowledge. Our final sample of health facilities was not fully nationally-representative of current ART service delivery characteristics in Uganda which limits the extent of generalizability of our study findings. However, our sample of facilities was derived from all the 10 geographic sub-regions of Uganda, had representation of the three major facility ownership-types in Uganda (public/for-profit/not-for-profit) and had representation from all three levels of care in the Ugandan health system (tertiary/secondary/primary). In addition, the study had other strengths including its mixed-methods research design which enabled us to ‘go beyond the numbers’. The qualitative findings offer explanatory insight into quantitative findings of trends in uptake of differentiated ART models in our sample of health facilities. Importantly, although much of the literature about differentiated ART delivery has come out of clinical trials, this study brings to light patient preferences of differentiated ART delivery at frontline points of service of delivery in Uganda.

## Conclusion

This is one of the first studies reporting the extent of uptake of differentiated ART delivery in a national sample of health facilities in Uganda. The majority of health facilities were implementing at least one less-intensive differentiated ART delivery model. Overall, facility-based models have had a higher rate of uptake in participating facilities. Barriers to uptake of community-based models include HIV-related stigma, insufficient funding and a preference by patients to see a health worker in person on a regular basis. Specific Interventions are also needed to promote the uptake of differentiated ART delivery in private-for-profit health facilities in Uganda which have the lowest coverage.

## Supporting information

S1 Annex Health facility survey. (DOCX)

S2 Annex (DOCX)
