# Supplementary material for: A mixed-methods evaluation of the uptake of novel differentiated ART delivery models in a national sample of health facilities in Uganda
Source: PLoS One. 2021 Jul 22;16(7):e0254214. doi: 10.1371/journal.pone.0254214 (PMC8297836; doi:10.1371/journal.pone.0254214)
Supplement: S1 Annex — (DOCX) [file pone.0254214.s001.docx]

**Annex A: Health facility survey**

Project title**: Understanding facilitators and barriers to the uptake of Differentiated Service Delivery (DSD) evidence in ART provision in health facilities in Uganda**

**INTRODUCTION**

Thank you for agreeing to meet us.

We are conducting a study looking at enablers and hindrances to the uptake of Differentiated Service Delivery (DSD) in anti-retroviral therapy (ART) services in health facilities in Uganda. We are interested in learning about facility-level facilitators and barriers to adoption or full implementation of DSD from the perspective of WHO’s six ‘building blocks’ (e.g. Human resources, financing, medicines and supplies etc.) Our goal is to provide practical information to local and national government, PEPFAR and other partners about how to increase scale-up of DSD models nationally and how to improve DSD for recipients of care. As part of the overall study, we are conducting facility surveys. We are conducting one cross sectional survey across the 10 geographic sub-regions of Uganda. The survey will cover service delivery, infrastructure and logistics support, supervision, drugs and commodities support, human resources, finance and budget, and other health systems aspects.

# section A: Facility Information

| *To be prefilled by enumerator but checked at site:*   - 1. Facility Name:   2. Facility District:   3. Facility level of care: 1. RRH 2. District Hospital 3. HC IV 4. HC III   4. Facility type: 1. Public 2. PNFP 3. PFP 4. Research site   5. 1. High Volume site 2. Low Volume site   6. Number of active ART clients …………………………………………………………. |
| --- |

- 1. **ART Clinic In-charge Characteristics:**

| 0.6.1 Name: |  |
| --- | --- |
| 0.6.2 Cadre: |  |
| 0.6.3 Highest level of Training: |  |
| 0.6.4 Date Became In-Charge at Current Facility: |  |
| 0.6.5 Age:  0.6.6 Male/Female |  |
| 0.6.6 Contact Information: |  |
| **0.7 Other Staff/Back-up Contact:** |  |
| 0.7.1 Name: |  |
| 0.7.2 Cadre: |  |
| 0.7.3 highest level of Training: |  |
| 0.7.4 dated started in current position at Facility: |  |
|  |  |
| 0.7.5 Age:  0.7.6 Male/female |  |
| 0.7.6 Contact Information: |  |

**SECTION B: EXTENT OF IMPLEMENTATION OF DSD MODELS AT FACILITY-LEVEL IN UGANDA**

1. Has your health facility implemented any aspect of Differentiated Service Delivery for ART?

1. YES 2. NO.

1. When did your health facility start implementing DSD, if at all?

1. 2016. 2. 2017 3. 2018 4. 2019 5. Not yet.

1. Which DSD model(s), if any, has your health facility implemented?

1. **CCLADS** (patient groups) 2. Fast-track refill (appointment spacing) 3. **CDDP** (community drug pick-ups) 4. FBIM (facility-based individualized models) 5. **FBG**: Facility based group 6………………………………..

1. a) What is your best estimate of the proportion of patients eligible for DSD at this facility who have been enrolled in DSD models?
2. All of them 2. Half of the patients 3. Two thirds 4. A third of them 5. Less than a third. 6. None of them.

What is the total number of patients enrolled in DSD in your facility……………………..

b) What is your best estimate of the proportion of patients enrolled in DSD models who have been retained in care? 1. All of them 2. Half of the patients 3. Two thirds 4. A third of them 5. Less than a third. 6. None of them.

1. In your experience, which DSD model is the most practical to implement at your facility given your operational context?

1. **CCLADS** (patient groups) 2. Fast-track refill (appointment spacing) 3. **CDDP** (community drug pick-ups) 4. FBIM (facility-based individualized models) 5. **FBG**: Facility based group 6………………………………..

1. In your experience, which of the two groups of DSD models have received more demand from clients at your facility?

1. Community-based models 2. Facility-based Models

1. Who currently assigns DSD models to which patients will be enrolled into?

1. Health worker 2. Client 3. Both (1 & 2).

1. Has your health facility made any modifications to the way DSD is implemented?

1. Yes 2. No

1. How would you rate the modifications made to DSD implementation?

1. No change. 2. Minor modifications. 3. Major modifications.

1. In your opinion what are the most important barriers to the uptake of community-based DSD models such as CCLADS (patient groups) or CDDP (community drug pick-ups)?
2. Stigma. 2. Lack of funding for implementation 3. Availability of guidance protocols

4. Low awareness of DSD models among clients 5………………………………………

**SECTION C: Facilitators and barriers to DSD evidence uptake at the facility-level**

Please indicate the degree to which you agree or disagree with the following statements, using a scale where “6” represents strongly agree and “1” represents strongly disagree.

| **WHO ‘building block’** | Strongly agree (6) | Agree (5) | | Somewhat agree (4) | | Somewhat disagree (3) | | Disagree (2) | Strongly disagree (1) | Not Applicable |
| --- | --- | --- | --- | --- | --- | --- | --- | --- | --- | --- |
| **Health Information systems** |  |  | |  | |  | |  |  |  |
| 1. The existing HMIS tools have been modified to support capture data for patients enrolled in DSD models. |  |  | |  | |  | |  |  |  |
| 2. The existing HMIS tools enable our staff to closely monitor patients enrolled in DSD models. |  |  | |  | |  | |  |  |  |
| 3. At this facility, we have the viral load data we need to differentiate/ assess patients for the DSD models they are eligible for. |  |  | |  | |  | |  |  |  |
| 4. Our TB patients attend their review appointments according to the DSD models in which they are enrolled. |  |  | |  | |  | |  |  |  |
| 5. Patients at this facility have adequate knowledge levels and awareness of Differentiated Service Delivery (DSD). |  |  | |  | |  | |  |  |  |
| **Human Resources for Health** |  |  | |  | |  | |  |  |  |
| 6. All health workers in the ART clinic have been trained in DSD for ART services. |  |  | |  | |  | |  |  |  |
| 7. All health workers in the ART clinic have adequate skills to competently offer DSD to our clients. |  |  | |  | |  | |  |  |  |
| 8.All health workers in our clinic are currently providing DSD in our ART services |  |  | |  | |  | |  |  |  |
| 9. Health workers in the ART clinic regularly attend refresher trainings in DSD service delivery. |  |  | |  | |  | |  |  |  |
| 10. Health workers in our ART clinic have readily available written guidelines or job aids on how to deliver DSD for ART. |  |  | |  | |  | |  |  |  |
| 11. Health workers at this clinic are able to accurately conduct patient differentiation for DSD. |  |  | |  | |  | |  |  |  |
| 12. Health workers in our ART clinic are sufficiently motivated in embracing DSD services. |  |  | |  | |  | |  |  |  |
|  | Strongly agree (6) | Agree (5) | | Somewhat agree (4) | | Somewhat disagree (3) | | Disagree (2) | Strongly disagree (1) | Not Applicable |
| 13. Health workers in our ART clinic are willing and ready to adopt Differentiated Service Delivery |  |  | |  | |  | |  |  |  |
| 14. Implementing DSD has reduced the workload of staff in the ART clinic. |  |  | |  | |  | |  |  |  |
| **Medicines and supplies** |  |  | |  | |  | |  |  |  |
| 15. Our facility is able to provide stable patients with a 3-month supply of ARVs. |  |  | |  | |  | |  |  |  |
| 16. We have experienced logistical challenges in providing 3-6 months supplies of ARVs to eligible clients. |  |  | |  | |  | |  |  |  |
| 17. Stock-outs are a frequent barrier to implementing DSD at this facility. |  |  | |  | |  | |  |  |  |
| 18. Patients who receive a 3-6 month supply of ARVs have been able to responsibly manage the stock in their homes. |  |  | |  | |  | |  |  |  |
| 19. We have adequate viral load laboratory supplies for making decisions on assigning DSD models for patients. |  |  | |  | |  | |  |  |  |
| **Governance and leadership** |  |  | |  | |  | |  |  |  |
| 20. The District Health Team provides regular support and monitoring of our DSD services. |  |  | |  | |  | |  |  |  |
| 21. The IP (implementing partner) provides continuous support supervision to our facility to promote DSD |  |  | |  | |  | |  |  |  |
| 22. The facility in-charge and the leadership of this facility have been supportive in enabling us provide DSD at this facility. |  |  | |  | |  | |  |  |  |
| 23. Our facility holds regular planning meetings for DSD services. |  |  | |  | |  | |  |  |  |
| 24. We have assigned a DSD focal person at this health facility. |  |  | |  | |  | |  |  |  |
| 25. We have a ‘program champion’ at our facility who actively advocates for DSD services. |  |  | |  | |  | |  |  |  |
| **Financing** |  |  | |  | |  | |  |  |  |
| 26. We draw upon the facility budget (e.g. PHC funds) to facilitate DSD implementation. |  |  | |  | |  | |  |  |  |
|  | Strongly agree (6) | Agree (5) | | Somewhat agree (4) | | Somewhat disagree (3) | | Disagree (2) | Strongly disagree (1) | Not Applicable |
| 27. Our Implementing Partner provides sufficient funding to support DSD uptake at this facility. |  |  | |  | |  | |  |  |  |
| 28. DSD implementation at this facility has transitioned from ‘pilot’ state to being a routine normal practice. |  |  | |  | |  | |  |  |  |
| 30. We have a dedicated funding source for implementing DSD at this facility. |  | |  | |  | |  |  |  |  |
| **Community health systems** |  | |  | |  | |  |  |  |  |
| 31. Our health facility has adequate funding for implementing community-based DSD models (e.g. CCLADS, CDDP). |  | |  | |  | |  |  |  |  |
| 32.We are able to monitor our patients enrolled in community-based DSD models |  | |  | |  | |  |  |  |  |
| 33. At this facility we have written guidelines on how to implement community-based DSD models. |  | |  | |  | |  |  |  |  |
| 34. We have written guidelines on how to implement community-based DSD models. |  | |  | |  | |  |  |  |  |
| 35.At this facility we have designated community ARVs pick-up points for clients in the CDDP model |  | |  | |  | |  |  |  |  |
| 36. At this facility we have devised strategies for combating stigma-related barriers to the uptake of community DSD models. |  | |  | |  | |  |  |  |  |
| 37. At this facility we have received ample demand from patients for community-based DSD models. |  | |  | |  | |  |  |  |  |
| 38. At this facility we have tapped into village health teams (VHTs) to support our community DSD models. |  | |  | |  | |  |  |  |  |

**ANNEX ‘B**

**Understanding facilitators and barriers to the uptake of Differentiated Service Delivery (DSD) evidence in ART provision in health facilities in Uganda**

**FOCUS GROUP DISCUSSION (FGD) GUIDE WITH PATIENTS ENROLLED IN DIFFERENTIATED SERVICE DELIVERY MODELS**

**DEMOGRAPHIC CHARACTERISTICS OF PARTICIPANTS** *(Interviewer to record the details below at the onset of the FGD and after explaining the objectives of the study)*

- Age
- Gender
- Length on ART
- DSD model of enrollment
- Level of Education

1. Please briefly tell me about yourself and how long you have been on ART.
2. Before the introduction of DSD models, how frequently did you attend this facility for reviews?
3. When and how did you come to be enrolled in a DSD model?
4. What is your comment on the level of patient awareness and sensitization on DSD services?
5. Please briefly tell me about the DSD model you are currently enrolled?
6. What advantages do you find in being in your current DSD model?

(*Probes: i) Savings in time ii) savings in transport costs iii) benefits of less time away from work iv) reduced congestion*

1. What challenges, if any, are you experiencing in the DSD model in which you are currently enrolled?

*(Probes: individual DSD models i) CCLAD ii) Fast-track refill (stock-outs) iii) FBIM iv) CDDP*

1. If you were to choose a DSD model that you prefer which would this be? Why do you prefer this particular model?
2. Differentiated Service Delivery is meant to be *patient-centred.* What is your comment on the extent to which current DSD implementation reflects your personal choices and preferences?
3. How have patients been involved in the development and implementation of DSD in your facility or community, if at all? *(Probes: i) development of treatment guidelines ii) SOPs iii) improving DSD models)*
4. How can patients be more involved in developing and improving DSD models?
5. In your opinion, why has the uptake of community DSD models not been as strong as intended?
6. What barriers to do you see to increased uptake of community-based DSD models? (*Probes patient literacy of DSD models, demand-creation campaigns prospects) inadvertent disclosure*
7. How important is stigma as a barrier to the uptake of community DSD models? Which strategies can be employed to combat stigma?
8. Why do patients tend to prefer facility-based DSD models? *Probes i) psychological care by a health worker ii) opportunity for comprehensive care*
9. How satisfied are you with the quality of care under the DSD model in which you are enrolled ?
10. Do you know any of your fellow patients who have declined to enroll into some DSD models or DSD altogether? Why did they opt against being enrolled into DSD model(s)?
11. How satisfied are you with the competence of health workers in providing DSD services?
12. Is there anything you want to tell us about DSD that we have not talked about?

**ANNEX ‘C**

**SEMI-STRUCTURED INTERVIEWS WITH HIV SERVICE MANAGERS (***National-level HIV program managers/District Health Team leaders / PEPFAR implementing organizations/national medicines supply chain managers)*

**INTERVIEW GUIDE**

**Preliminary Questions**

1. Please tell me a little about yourself and what you do in this role?

How long have you been in this position?

2. What your personal assessment is of how well are we doing as a country in implementing DSD nationally?

3. In your opinion, what are the successes registered so far in implementing DSD nationally?

4. What are the challenges standing in the way of further scale-up of DSD models nationally?

*(Interviewer, based on respondents answers to questions* ***3*** *and* ***4*** *skip some of the questions posed below)*

**Pharmaceutical supply chains**

5. There have been press reports of stock-outs of ARVs. How has this affected the multi-month dispensing required under DSD, if at all?

6. Uganda’s centralized medicines supply chain system is based on bi-monthly orders. How has this affected the new demands of 3-6 month supplies of ARVs?

(*Probes: NMS warehouse capacity, storage capacities at facility-level and households of clients).*

7. What strategies are needed to ensure we have sufficient stock of HIV medicines in the context of DSD?

Which policy shifts are necessary to align with the new demands of multi-month dispensing under DSD?

**Enabling policies and supporting national guidelines**

8. How have the current HMIS tools hindered or facilitated DSD implementation especially at facility-level?

(*Probes: Do current tools allow for DSD monitoring and evaluation, program reporting, patient follow-up, alignment with the multiple DSD models?)*

9. What is your comment on the clash between DSD appointment spacing and monthly TB reviews?

*(Probes: Is there a need for updating Guidelines?)*

10. Early implementation experiences suggest that there has been especially low uptake of community- based DSD models. In your opinion, why is the case?

(*Probes: Guidelines for community implementation, stigma (policy and strategies), financing, leveraging existing community health systems such as VHTs)*

11. How have stable patients in community-based DSD models been monitored by health facilities and what can be done to improve follow-up under DSD?

(*Probes: updating HMIS tools? developing guidelines on follow-up in community models?)*

**Community health-systems**

12. What strategies are needed to increase the funding available to health facilities to scale-up community-based DSD models?

13. How has stigma affected the roll-out of community-based DSD models, if at all?

*(Probes: strategies for combating stigma)*

14. What role do you envisage of demand-creation campaigns in the further scale-up of community-based DSD models?

*(Probes: leveraging*

**Health workforce**

15. In what ways have health workforce factors facilitated or hinder the national implementation of DSD? *(Probes: Health worker DSD competence (knowledge and attitudes), current state of health worker training roll-out/ workload issues)*

16. What policies have been developed to enable the task-shifting approaches involved in DSD models? (*Such as in empowering clients in managing peers through CCLADS and CDDP?)*

**Sub-national implementation**

17. What factors have hindered or enabled the implementation of DSD at district- level?

*(Probes: buy-in from leadership/ district budget support/ DHT support supervision)*

Has DSD implementation received adequate support from the political and administrative leadership of districts?

18. From the perspective of implementing partners (IPs), what facilitators and barriers have you encountered in supporting health facilities to adopt DSD?

*(Probes: donor policies (geographic prioritization)/ budgets/ district support)*

**Financing**

19.How have health financing issues facilitated or hindered the national roll-out of DSD models?

(*Probes: National budget support/ adequacy of donor funding/ sub-national level funding).*

**Appendix 1. 1 Consent form for ART clinic in-charges taking part in the health facility survey.**

**Understanding the facilitators and barriers to the uptake of Differentiated Service Delivery (DSD) evidence for ART services in health facilities in Uganda**

**Introduction**

Globally, there is growing consensus that traditional ‘one-size-fits-all’ models of clinic-based, physician-centred HIV care are unsustainable in resource-constrained settings given the escalating demand for anti-retroviral therapy (ART) and declining international assistance. Differentiating HIV care to the needs of individual patients (such as spacing appointments, pharmacy-only refills for stable patients) is critical.

Although there is accumulating experimental evidence demonstrating that Differentiated Service Delivery (DSD) reduces the costs of HIV services delivery, decreases pressure on over-burdened African health systems and results in better patient outcomes, there has been limited uptake at the policy and operational-level in countries with a high HIV burden including Uganda. Despite the fact that Uganda released national ART treatment guidelines in 2016 recommending DSD, it is estimated that only 30% of health facilities are implementing DSD contrary to World Health Organization recommendations.

**Purpose of the Study**

The aim of this study is to understand facilitators and barriers to the uptake of Differentiated Service Delivery (DSD) evidence in policy and practice by diverse stakeholders in the health system in Uganda. In this study we pose this question: *What are the facilitators and barriers to the uptake of DSD evidence in policy and practice in Uganda and how can we accelerate scale-up?*

**Study Procedures**

You have been selected because of your influential role in HIV service delivery at the facility-level in Uganda. We seek to draw upon your personal experience and knowledge of your operational context to inform this study. If you accept to participate in this study, we will seek to understand your perspective on the factors facilitating or hindering the uptake of DSD evidence in ART services at the facility-level in Uganda. A researcher-administered questionnaire will be fielded to you by a member of our research team. We expect that completing the survey will to take 40 to 60 minutes.

**Potential Benefits**

There is no direct benefit to you for taking part in the study. However, the results of this study will help policy-makers, planners and service providers to improve the quality of HIV services and patient outcomes for those enrolled on ART as well reduce overcrowding in ART sites across Uganda and reduce health worker workloads.

**Potential Risks**

We do not anticipate major risks from this study. However, in case you feel uncomfortable at any time, you do not have to answer the questions and you may stop at any time. Your name will not be used in any reports or publications of research. Records of your interview will be kept confidential.

**Voluntary Participation and Withdraw**

Your participation in this study is voluntary. You are free to withdraw anytime, and you will not be required to give a reason for your decision. Also note that your choice to participate or not to participate in this study will not affect you in any way.

**Statement of Confidentiality**

Any information you share with us will only be shared among the researchers on this study and will be kept confidential. Your name or any other information that could identify you will not be used in any research records, reports or publications from this study. Audio file and notes will not contain any personal identifiers of participants and will be assigned a unique identifier to keep your identity confidential.

**Costs of taking part in this study**

You will not pay anything to take part in this study and you will not be paid for taking part in the study. Apart from your time, you will not incur any other costs when you participate in the study. You will be paid 20,000 Uganda Shillings as transport refund.

**Contact Information for Questions or Concerns**

In case you have any questions or concerns regarding the study you may contact the following:

Dr. Henry Zakumumpa, PI, Tel. +256-772-520519, Email: zakumumpa@covab.mak.ac.ug

For questions regarding your rights or any other ethical issues, you may contact the

Chairperson Mildmay Uganda Research Ethics Committee (MUREC): Harriet Chemusto (0392174236) Email: [murec@mildmay.org.ug](mailto:murec@mildmay.org.ug)

#### Consent and Signature

The study has been explained to me and my questions/concerns about the study have been answered to my satisfaction. I voluntarily agree to participate in this study.

___________________________________________________________________________

Print name of Participant Signature of Participant Date

___________________________________________________________________________

Print name of Person Obtaining Signature of Person Obtaining Consent Date

Consent

**Appendix 1. 2 Consent form for patients taking part in the Focus Group Discussions (FGDs)**

**Understanding the facilitators and barriers to the uptake of Differentiated Service Delivery (DSD) evidence for ART services in health facilities in Uganda**

**Introduction**

Globally, there is growing consensus that traditional ‘one-size-fits-all’ models of clinic-based, physician-centred HIV care are unsustainable in resource-constrained settings given the escalating demand for anti-retroviral therapy (ART) and declining international assistance. Differentiating HIV care to the needs of individual patients (such as spacing appointments, pharmacy-only refills for stable patients) is critical.

Although there is accumulating experimental evidence demonstrating that Differentiated Service Delivery (DSD) reduces the costs of HIV services delivery, decreases pressure on over-burdened African health systems and results in better patient outcomes, there has been limited uptake at the policy and operational-level in countries with a high HIV burden including Uganda. Despite the fact that Uganda released national ART treatment guidelines in 2016 recommending DSD, it is estimated that only 30% of health facilities are implementing DSD contrary to World Health Organization recommendations.

**Purpose of the Study**

The aim of this study is to understand facilitators and barriers to the uptake of Differentiated Service Delivery (DSD) evidence in policy and practice by diverse stakeholders in the health system in Uganda. In this study we pose this question: *What are the barriers to the uptake of DSD evidence in policy and practice in Uganda and how can we accelerate scale-up?*

**Study Procedures**

You have been selected because of you have experience as a recipient of care enrolled in Differentiated ART service delivery and have personal insights to share about the particular DSD model in which you are enrolled. If you accept to participate in this study, we will seek to understand your personal experiences of being under DSD and your preferences and opinions on how DSD has worked for you (or not) and how DSD can be improved. You will participate in a group discussion with other recipients of care (6-8). We anticipate the focus group discussion to last between 45-60 minutes.

**Potential Benefits**

There is no direct benefit to you for taking part in the study. However, the results of this study will help policy-makers, planners and service providers to improve the quality of HIV services and patient outcomes for those enrolled on ART as well reduce congestion and overcrowding in ART sites across Uganda.

**Potential Risks**

We do not anticipate major risks from this study. However, in case you feel uncomfortable at any time, you do not have to answer the questions and you may stop at any time. Your name will not be used in any reports or publications of research. Records of your interview will be kept confidential.

**Voluntary Participation and Withdraw**

Your participation in this study is voluntary. You are free to withdraw anytime, and you will not be required to give a reason for your decision. Also note that your choice to participate or not to participate in this study will not affect you in any way.

**Statement of Confidentiality**

Any information you share with us will only be shared among the researchers on this study and will be kept confidential. Your name or any other information that could identify you will not be used in any research records, reports or publications from this study. Audio file and notes will not contain any personal identifiers of participants and will be assigned a unique identifier to keep your identity confidential.

**Costs of taking part in this study**

You will not pay anything to take part in this study and you will not be paid for taking part in the study. Apart from your time, you will not incur any other costs when you participate in the study. You will be paid 20,000 Uganda Shillings as transport refund.

**Contact Information for Questions or Concerns**

In case you have any questions or concerns regarding the study you may contact the following:

Dr. Henry Zakumumpa, PI, Tel. +256-772-520519, Email: zakumumpa@covab.mak.ac.ug

For questions regarding your rights or any other ethical issues, you may contact the

Chairperson Mildmay Uganda Research Ethics Committee (MUREC): Harriet Chemusto (0392174236) Email: [murec@mildmay.org.ug](mailto:murec@mildmay.org.ug)

#### Consent and Signature

The study has been explained to me and my questions/concerns about the study have been answered to my satisfaction. I voluntarily agree to participate in this study.

___________________________________________________________________________

Print name of Participant Signature of Participant Date

___________________________________________________________________________

Print name of Person Obtaining Signature of Person Obtaining Consent Date

Consent

**Appendix 1. 3 Consent form for HIV program managers and policy makers taking part in semi-structured interviews**

**Understanding the facilitators and barriers to the uptake of Differentiated Service Delivery (DSD) evidence for ART services in health facilities in Uganda**

**Introduction**

Globally, there is growing consensus that traditional ‘one-size-fits-all’ models of clinic-based, physician-centered HIV care are unsustainable in resource-constrained settings given the escalating demand for anti-retroviral therapy (ART) and declining international assistance. Differentiating HIV care to the needs of individual patients (such as spacing appointments, pharmacy-only refills for stable patients) is critical.

Although there is accumulating experimental evidence demonstrating that Differentiated Service Delivery (DSD) reduces the costs of HIV services delivery, decreases pressure on over-burdened African health systems and results in better patient outcomes, there has been limited uptake at the policy and operational-level in countries with a high HIV burden including Uganda. Despite the fact that Uganda released national ART treatment guidelines in 2016 providing for DSD services, it is estimated that only 30% of health facilities are implementing DSD contrary to World Health Organization recommendations.

**Purpose of the Study**

The aim of this study is to understand facilitators and barriers to the uptake of Differentiated Service Delivery (DSD) evidence in policy and practice by diverse stakeholders in the health system in Uganda. In this study we pose this question: *What are the barriers to the uptake of DSD evidence in policy and practice in Uganda and how can we accelerate scale-up?*

**Study Procedures**

You have been selected because of your influential role in HIV services delivery either at the programming or policy-maker level in Uganda. If you accept to participate in this study, we will seek to understand your perspective on the factors facilitating or hindering the uptake of DSD evidence in policy and practice in Uganda based on your professional experience and individual perspective. We expect that this interview will take 30 to 45 minutes. The interview will be audio recorded to enable the research team to capture all proceedings to allow the research term to understand what is emerging as study findings.

**Potential Benefits**

There is no direct benefit to you for taking part in the study. However, the results of this study will help policy-makers, planners and service providers on how improve the quality of HIV services and patient outcomes for those enrolled on ART as well reduce congestion and overcrowding in ART sites across Uganda.

**Potential Risks**

We do not anticipate major risks from this study. However, in case you feel uncomfortable at any time, you do not have to answer the questions and you may stop at any time. Your name will not be used in any reports or publications of research. Records of your interview will be kept confidential.

**Voluntary Participation and Withdraw**

Your participation in this study is voluntary. You are free to withdraw anytime, and you will not be required to give a reason for your decision. Also note that your choice to participate or not to participate in this study will not affect you in any way.

**Statement of Confidentiality**

Any information you share with us will only be shared among the researchers on this study and will be kept confidential. Your name or any other information that could identify you will not be used in any research records, reports or publications from this study. Audio file and notes will not contain any personal identifiers of participants and will be assigned a unique identifier to keep your identity confidential.

**Costs of taking part in this study**

You will not pay anything to take part in this study and you will not be paid for taking part in the study. Apart from your time, you will not incur any other costs when you participate in the study. You will be paid 20,000 Uganda Shillings as transport refund.

**Contact Information for Questions or Concerns**

In case you have any questions or concerns regarding the study you may contact the following:

1. Dr. Henry Zakumumpa, PI, Tel.+256-772-520519, Email: zakumumpa@covab.mak.ac.ug

For questions regarding your rights or any other ethical issues, you may contact the

Chairperson Mildmay Uganda Research Ethics Committee (MUREC): Harriet Chemusto (0392174236) Email: [murec@mildmay.org.ug](mailto:murec@mildmay.org.ug)

#### Consent and Signature

The study has been explained to me and my questions/concerns about the study have been answered to my satisfaction. I voluntarily agree to participate in this study.

___________________________________________________________________________

Print name of Participant Signature of Participant Date

___________________________________________________________________________

Print name of Person Obtaining Signature of Person Obtaining Consent Date

Consent

Dr Henry Zakumumpa

Makerere University,

College of Humanities and Social Sciences (CHUSS)

Office of the Principal,

Kampala, Uganda

August 2019

The Chairperson,

Mildmay Uganda Research Ethics Committee (MUREC),

Re: Request for ethical review of our Proposal for a Study titled- **Understanding facilitators and barriers to the uptake of Differentiated Service Delivery (DSD) evidence in ART provision in health facilities in Uganda**

This is to formally apply for ethical review of the above-named proposal. We seek to conduct a mixed-methods study to examine facilitators and barriers to the uptake of DSD evidence in anti-retroviral (ART) provision in health facilities in Uganda.

This study entails three components a) a health facility survey b) Focus group discussions with patients enrolled in DSD models and c) Semi-structured interviews with HIV service managers and policy-makers.

We propose to conduct this study in 10 districts of Uganda Mbarara (Southwest), Gulu (North), Tororo (Eastern), Jinja (East Central), Arua (West Nile), Hoima (Western), Masaka (Central 1), Mityana (Central 2), Napak (Karamoja) and Kampala (Kampala).

This study is conducted as part of a post-doctoral research project (supporting letters attached) and has been reviewed by an international panel of reviewers commissioned by African Population and Health Research Centre based in Nairobi, Kenya (<https://aphrc.org/>).

Your support will be highly appreciated to enable timely completion of the above study to inform efforts for the further scale-up of DSD nationally in Uganda.

Thank you for your assistance.

Yours,

Dr Henry Zakumumpa

Principal Investigator

Email: zakumumpa@yahoo.com, Tel: 0772-520519

**Proposal format**

**Introduction**

1. Background to the study
2. Statement of the problem
3. Justification of the study
4. Conceptual framework
5. Research questions
6. Objective of the study
7. Literature review

**Methodology**

1. Study design
2. Study areas
3. Study population
4. Sample size calculation
5. Sampling procedures
6. Study variables
7. Data collection
8. Field work procedures
9. Training of research assistants
10. Tools
11. Pre-testing
12. Field editing of data
13. Missing data
14. Data management and analysis
15. Data entry and cleaning
16. Analysis plan and dissemination plan
17. Ethical considerations

**Appendices**

1. Consent forms
2. Work plan and Budget
