# Supplementary material for: A mixed-methods evaluation of the uptake of novel differentiated ART delivery models in a national sample of health facilities in Uganda
Source: PLoS One. 2021 Jul 22;16(7):e0254214. doi: 10.1371/journal.pone.0254214 (PMC8297836; doi:10.1371/journal.pone.0254214)
Supplement: S2 Annex — (DOCX) [file pone.0254214.s002.docx]

**ANNEX B**

**FOCUS GROUP DISCUSSION (FGD) GUIDE WITH PATIENTS ENROLLED IN DIFFERENTIATED SERVICE DELIVERY MODELS**

**DEMOGRAPHIC CHARACTERISTICS OF PARTICIPANTS** *(Interviewer to record the details below at the onset of the FGD and after explaining the objectives of the study)*

- Age
- Gender
- Length on ART
- DSD model of enrollment
- Level of Education

1. Please briefly tell me about yourself and how long you have been on ART.
2. Before the introduction of DSD models, how frequently did you attend this facility for reviews?
3. When and how did you come to be enrolled in a DSD model?
4. What is your comment on the level of patient awareness and sensitization on DSD services?
5. Please briefly tell me about the DSD model you are currently enrolled?
6. What advantages do you find in being in your current DSD model?

(*Probes: i) Savings in time ii) savings in transport costs iii) benefits of less time away from work iv) reduced congestion*

1. What challenges, if any, are you experiencing in the DSD model in which you are currently enrolled?

*(Probes: individual DSD models i) CCLAD ii) Fast-track refill (stock-outs) iii) FBIM iv) CDDP*

1. If you were to choose a DSD model that you prefer which would this be? Why do you prefer this particular model?
2. Differentiated Service Delivery is meant to be *patient-centred.* What is your comment on the extent to which current DSD implementation reflects your personal choices and preferences?
3. How have patients been involved in the development and implementation of DSD in your facility or community, if at all? *(Probes: i) development of treatment guidelines ii) SOPs iii) improving DSD models)*
4. How can patients be more involved in developing and improving DSD models?
5. In your opinion, why has the uptake of community DSD models not been as strong as intended?
6. What barriers to do you see to increased uptake of community-based DSD models? (*Probes patient literacy of DSD models, demand-creation campaigns prospects) inadvertent disclosure*
7. How important is stigma as a barrier to the uptake of community DSD models? Which strategies can be employed to combat stigma?
8. Why do patients tend to prefer facility-based DSD models? *Probes i) psychological care by a health worker ii) opportunity for comprehensive care*
9. How satisfied are you with the quality of care under the DSD model in which you are enrolled ?
10. Do you know any of your fellow patients who have declined to enroll into some DSD models or DSD altogether? Why did they opt against being enrolled into DSD model(s)?
11. How satisfied are you with the competence of health workers in providing DSD services?
12. Is there anything you want to tell us about DSD that we have not talked about?
